# Why not see a doctor when ill? Evidence from the Chinese elderly

**DOI:** 10.1186/s12913-019-4212-0

**Published:** 2019-06-10

**Authors:** Shangren Qin, Ye Ding

**Affiliations:** 1 Hangzhou Normal University, 2318 Yuhangtang Road, Hangzhou, 311121 China; 2 Department of Public Health, Hangzhou Medical College, 481 Binwen Road, Hangzhou, 310053 China

**Keywords:** China, Not see a doctor, Unmet need

## Abstract

**Background:**

Elder people aged ≥45 years often have more healthcare needs than the younger. But the Chinese elderly are less likely to see a doctor when ill. In this article, this phenomenon is abbreviated as “not see a doctor”. This study aimed to describe the reason distribution of“not see a doctor” among the Chinese elderly. Specifically,we examined the reasons why“not see a doctor” happened to the Chinese elderly with different characteristics.

**Methods:**

In order to explore the associations between various predisposing, enabling and need factors and “not see a doctor” in China, this cross-sectional study used the data from the 2015 wave 4 of the China Health and Retirement Longitudinal Study (CHARLS). Using multivariate analyses, associations between “not see a doctor” and factors were accessed. Models were estimated using a binary model with negative log-log link function (cases versus controls) and multinomial logit analysis (reasons for “not see a doctor”).

**Results:**

Adjusted by individual weight, the analysis included 16,277 people aged ≥45 years, of whom 11% reported “not see a doctor”. Overall, those with older age, other marital status (except married) and poorer health status were more likely to report “not see a doctor”. No significant associations were found between income and “not see a doctor”. The majority of cases report “no need” as the reason for their “not see a doctor”. Except reason “no need”, factor associated with the healthcare system—cost—accounted for the most case of “not see a doctor”. Those without health insurance are more likely not to see a doctor due to affordability issues.

**Conclusions:**

This quantitative study suggests that “not see a doctor” is more likely to happen due to age and marital status issues, especially affordability issues. For China, it is important to enforce the policy of reducing of healthcare fees and increasing health insurance coverage.

## Background

China has witnessed the increasing elderly population in recent years. Specifically, according to China Statistical Yearbook, the population aged ≥45 years increased 18%, from 473.8 million in 2012 to 558.9 million in 2017.

Compared with the younger adults, the elderly people often have higher demands for health care, which can be ascribed to the decline in physical function and increase in age-related diseases [1]. In addition, retirement and the decreased income are likely to result in a less financially secure environment. The spending on health and social care have brought growing burdens on both the families and the individuals [2]. Therefore, the elderly people would probably not see a doctor when they are ill. In this article, this phenomenon was abbreviated as “not see a doctor”.

“Not see a doctor” is different from “unmet healthcare demand”. Concretely, unmet healthcare demand is defined as a measure of “the differences, if any, between services that are judged necessary to appropriately deal with defined health problems and those that are actually received” [3]. In short, unmet healthcare demand suggests that “someone really and necessarily needs a medical examination or treatment for a health problem but he doesn’t receive it”; while the definition of “not see a doctor” does not emphasize the necessity of healthcare demand.

In China, the phenomenon of “not see a doctor” among elderly people is also present. About 17.67% of elderly people aged ≥45 years in China’s Guangdong Province didn’t go to see a doctor when they were ill [4]. This rate was 41.7% among caregivers (88.4% of them aged ≥40 years) of left-behind children in poor rural areas of China’s Hunan Province [5]. However, for the whole country, there is little data on “not see a doctor” among elderly people.

Chinese policy makers have long been struggling to try best to solve the problem of “not see a doctor” among the elderly. However, few theoretical models are available to explain the necessity of healthcare, which may result in the inappropriate utilization of healthcare services [6]. Typically, a useful starting point is Andersen’s behavioral model of health services [7], which focuses on predisposing, enabling, and demand factors assumed to be associated with a person’s use of health services.

Using the Andersen’s model, a few factors, including sex, age and health status, have been found to be associated with unmet healthcare demand, yet some associations are inconsistent among different studies. For example, some studies find higher unmet healthcare demands among the female population [6, 8], while others have discovered no significant relationship [9]. Nonetheless, studies have consistently found higher unmet healthcare demands among the population with poorer health status and higher economic burdens [10].

However, these associated factors are found in other countries [11, 12], and research on the unmet healthcare demand and on “not see a doctor” is limited in China. Therefore, this study aimed to explore the associations of various predisposing, enabling and need factors with “not see a doctor” in China.

## Methods

### Design of the study

This study was a cross-sectional study to analyze the specific population who do not see a doctor when ill and the main reasons why they don’t seek medical treatment. The data was from 2015 wave 4 of the China Health and Retirement Longitudinal Study (CHARLS) and the study population was elderly Chinese aged ≥45 years. Firstly, the study described the status of “not see a doctor” among elderly people. Using the Andersen’s model and a binary model with negative log-log link function, susceptible people who did not go to see a doctor when ill were explored. Secondly, the main reasons for “not see a doctor” were investigated. The associations between the main reasons and factors of Andersen’s model were evaluated using a multinomial logistic regression.

### Data

Data in this study were derived from the 2015 wave 4 of CHARLS. Generally, CHARLS, which was conducted by the China Centre for Economic Research of Peking University, was a nationally representative survey on the non-institutionalized adults aged ≥45 in mainland China. In the database of CHARLS in 2015, there were a total of 21,100 individuals, in which 369 were missing age and 1013 were under 45 years. As a result, there were 19,718 individuals aged ≥45 years, in which 3441 didn’t answer the question whether they went to see a doctor in the last month or not. Finally, 16,277 individuals were included in our study.

Information of the respondents, including socioeconomic status, demographic characteristics, health-related behaviors and lifestyles, health status (such as health insurance, health conditions and health services use) were collected, through face-to-face interview. More details on the sampling method, the questionnaire and quality assurance measures were available from Zhao et al. [13], and the CHARLS data could be downloaded at the official website (charls.pku.edu.cn/en).

### Andersen’s behavioral model of health services

The Andersen’s model, developed in 1968, has been widely applied in numerous studies on health services. It hypothesizes that the use of health services in a person is a function of several factors, such as the predisposing, enabling and demand factors, among which, the predisposing factors include socio-demographic variables (such as age, sex, education and marital status), while the enabling factors refer to resources that can impede or facilitate the use of health services (namely, income, access to health insurance or pension and wealth), and demand factors represents the demand for health care services, and is generally related to health status.

### Measures

The self-reported “not see a doctor” was a dependent variable measured by the two following questions included in CHARLS: (1). Have you been ill in the last month? (2). In the last month, have you visited a public hospital, private hospital, public health center, clinic, or health worker’s or doctor’s practice, or been visited by a health worker or doctor for outpatient care? Typically, those who answered “Yes” to question 1 and “No” to question 2 simultaneously were recognized as the cases; otherwise, they were deemed as the controls. The cases were then asked to answer the main reasons of “not see a doctor” when ill and choose the main reason based on subjective feelings.

Similar to other studies [6, 14], several common variables were selected as the study factors in this study. Specifically, the predisposing factors included sex, age, marital status, hukou (household registration) and education level. The hukou system in China is an institution that controls population mobility, in which an individual is required to register in one and only one place of regular residence. Typically, the most common classification of hukou is rural hukou and urban hukou [15]. As for enabling factors, they included total annual individual income, as well as the access to health insurance and pension. The total annual individual income was calculated as the sum of annual individual income only from four sources (wage, bonus income, pensions and government subsidy), and per capita annual household income from all sources (e.g. income from all crops and forestry products, and income from all livestock and aquatic life). Afterwards, the total annual individual income was divided into tertiles according to the household register, with one third of the study population in each group. Moreover, the demand factors in the study were the self-reported general health status and chronic illness.

### Statistical analysis

The prevalence of “not see a doctor” was described after adjusted by the individual weight. To be specific, the individual weight in CHARLS denoted the inverse of the probability that the subject was included because of the sampling design and the adjustment with individual and household non-response [16].

One of the dependent variables was “not see a doctor”. The probability of “not see a doctor” was small and the negative log-log link mode was needed. A binary model with negative log-log link function was implied to evaluate the associations between “not see a doctor” and predisposing, enabling as well as demand factors. The other dependent variables was “reasons for not see a doctor”. A multinomial logistic regression was used to access the associations between “reasons for not see a doctor” and related factors. Odds Ratio (OR) and its 95% confidence interval (95%CI) were calculated.

All statistical tests were 2-sided, and *P*-values of < 0.05 were considered as statistically significant. Data were statistically analyzed using SPSS version 22.

## Results

A total of 16,277 subjects aged ≥45 were enrolled in this analysis, including 1844 (11%) who reported “not see a doctor”. Table 1 showed the distribution of weighted proportion of subjects reporting “not see a doctor” according to the predisposing, enabling and demand factors. For the predisposing factors, a higher proportion of subjects with older age, other marital status and lower education level had reported a higher rate of “not see a doctor”. With regard to the enabling factors, those with lower income seemed to have reported a higher level of “not see a doctor”. As for the need factors, subjects with poor health status and more chronic diseases had reported a higher level of “not see a doctor”.

**Table 1:** Associations between factors and “not see a doctor” among Chinese aged≥45 years, 2015. (N (weighted, %))

Factors	Control group^a^	“Not see a doctor” group^a^	OR(95%CI)^b^
Sex			
Female	7170 (87.72)	1023 (12.28)	Reference
Male	7263 (89.57)	821 (10.43)	0.963 (0.906–1.024)
Age group			
75+	1430 (85.72)	197 (14.28)	Reference
60–74	5782 (88.99)	741 (11.01)	1.125 (1.018–1.243)^*^
45–59	7221 (89.02)	906 (10.98)	1.253 (1.124–1.397)^***^
Marital status			
Married	11,761 (89.38)	1410 (10.62)	Reference
Others^c^	2672 (85.69)	434 (14.31)	1.092 (1.014–1.176)^*^
Hukou			
Rural resident	10,048 (88.04)	1355 (11.96)	Reference
Urban resident	2901 (89.47)	326 (10.53)	0.974 (0.903–1.052)
Education			
No formal	2632 (87.28)	375 (12.72)	Reference
Elementary school	3799 (88.02)	550 (11.98)	1.016 (0.944–1.093)
Middle school and above	3186 (89.90)	324 (10.10)	0.939 (0.861–1.025)
Income			
Low	5035 (87.28)	644 (12.72)	Reference
Middle	4314 (88.02)	613 (11.98)	1.060 (0.986–1.141)
High	4659 (89.90)	543 (10.10)	1.073 (0.997–1.154)
Access to pension^d^			
No	3935 (88.83)	522 (11.17)	Reference
Yes	10,488 (88.57)	1319 (11.43)	0.971 (0.909–1.037)
Access to health insurance^e^			
No	1277 (87.72)	173 (12.28)	Reference
Yes	13,156 (88.73)	1671 (11.27)	0.943 (0.853–1.042)
Self-reported general health status			
Bad	1578 (78.30)	425 (21.70)	Reference
Fair	4622 (87.03)	704 (12.97)	0.811 (0.746–0.883)^***^
Very good/good	7455 (92.56)	585 (7.44)	0.660 (0.606–0.719)^***^
Chronic illness			
No chronic disease	3792 (93.07)	265 (6.93)	Reference
One chronic disease	3071 (91.99)	266 (8.01)	1.102 (1.012–1.200)^*^
Two chronic diseases	2124 (90.71)	230 (9.29)	1.161 (1.061–1.270)^**^
Three and more chronic diseases	2836 (80.67)	671 (19.33)	1.548 (1.423–1.683)^***^

^a^All proportions are weighted

^b^The binary model with negative log log link is not weighted. *P < 0.05, ^**^P < 0.01, ^***^P < 0.001

^c^Include divorced or separated, widowed and single

^d^Include rural pension, urban residents’ pension, commercial pension, pension of the firms, pension program of the government or institutions and old age pension allowance or others

^e^Include urban employee medical insurance, urban and rural resident medical insurance, new cooperative medical insurance, urban resident medical insurance, private medical Insurance, government medical insurance

Table 1 also displayed the associations between factors and “not see a doctor”. As could be observed from a binary regression with negative log-log link function, those with younger age were more likely to report “not see a doctor” than those aged > 75 years. In addition, compared with the married subjects, those with other marital status were markedly associated with a higher odds of reporting “not see a doctor”. Subjects with poorer health or more chronic diseases would also reported a notably higher level of “not see a doctor”. However, no significant associations were found between enabling factors and “not see a doctor”.

Table 2 presented the distribution of reasons for “not see a doctor” among all factors. As could be observed, a majority of cases reported no need as the reason for their “not see a doctor”, since they thought that their illness was not serious and thereby did not need to see a doctor. Besides, those with rural hukou had reported a higher proportion of affordability issue as the major cause compared with those with urban hukou. While those with better health status were less likely to report the cost issues as the major cause relative to those with poorer health. Additionally, among those without either pension or health insurance, affordability issue was one of the main reasons for “not see a doctor”.

**Table 2:** Distribution of reasons for “not see a doctor” among Chinese aged≥45 years, 2015.(N (weighted, %))

Factors	No need	Inconvenient traffic or no time to see doctor	Distrust doctors or hospital service	Could not afford	Other
Sex					
Female	675 (63.59)	69 (5.85)	48 (4.29)	187 (15.79)	124 (10.48)
Male	575 (65.55)	44 (4.96)	44 (5.10)	128 (13.49)	81 (10.90)
Age group					
75+	109 (53.40)	19 (7.39)	17 (5.34)	35 (13.12)	33 (20.74)
60–74	475 (60.39)	43 (5.77)	39 (4.67)	137 (16.54)	103 (12.64)
45–59	666 (70.97)	51 (4.62)	36 (4.44)	143 (13.83)	69 (6.14)
Marital status					
Married	989 (67.89)	79 (4.99)	70 (4.58)	237 (14.04)	143 (8.49)
Others^a^	261 (54.21)	34 (6.80)	22 (4.88)	78 (16.91)	62 (17.20)
Hukou					
Rural	895 (61.55)	92 (6.05)	69 (5.30)	253 (16.30)	156 (10.80)
Urban	240 (73.70)	13 (4.21)	17 (3.86)	32 (10.05)	33 (8.17)
Education					
No formal	218 (53.49)	29 (6.75)	14 (3.06)	80 (20.07)	65 (16.63)
Elementary school	373 (62.52)	27 (4.95)	27 (4.70)	105 (17.22)	56 (10.61)
Middle school and above	245 (73.11)	16 (3.84)	15 (4.22)	39 (10.70)	32 (8.14)
Income					
Low	436 (64.24)	43 (6.40)	34 (5.36)	110 (15.35)	68 (8.65)
Middle	383 (58.93)	37 (5.19)	30 (4.09)	123 (18.49)	80 (13.30)
High	398 (70.29)	31 (4.78)	28 (4.81)	70 (9.13)	55 (10.99)
Access to pension^b^					
No	319 (57.51)	28 (4.45)	29 (5.42)	114 (22.37)	63 (10.25)
Yes	931 (67.18)	84 (5.75)	62 (4.38)	200 (11.86)	142 (10.84)
Access to health insurance^c^					
No	101 (58.00)	8 (3.76)	10 (4.51)	46 (22.78)	20 (10.96)
Yes	1149 (65.19)	105 (5.63)	82 (4.67)	269 (13.88)	185 (10.63)
Self-reported general health status					
Bad	218 (51.76)	22 (4.16)	31 (6.10)	124 (26.34)	58 (11.65)
Fair	496 (66.46)	40 (4.75)	27 (4.41)	112 (15.36)	72 (9.01)
Very good/good	457 (72.85)	40 (6.17)	28 (4.49)	64 (8.52)	53 (7.97)
Chronic illness					
No chronic disease	211 (74.89)	19 (5.69)	10 (3.32)	28 (9.36)	24 (6.74)
One chronic disease	214 (74.10)	13 (3.80)	8 (3.87)	30 (9.92)	22 (8.32)
Two chronic diseases	167 (65.46)	14 (5.81)	9 (5.33)	35 (14.38)	25 (9.02)
Three and more chronic diseases	376 (55.49)	42 (5.41)	41 (4.80)	152 (19.39)	94 (14.90)

^a^Include divorced or separated, widowed and single

^b^Include rural pension, urban residents’ pension, commercial pension, pension of the firms, pension program of the government or institutions and old age pension allowance or others

^c^Include urban employee medical insurance, urban and rural resident medical insurance, new cooperative medical insurance, urban resident medical insurance, private medical Insurance, government medical insurance

Table 3 had displayed the adjusted odds ratios (ORs) from a multivariate multinomial logistic regression of reasons for “not see a doctor”. Notably, the characteristics of those reporting “not see a doctor” due to “inconvenient traffic or no time to see doctor”, “distrust doctors or hospital service”, “could not afford” and “other” reasons were compared to those reporting “not see a doctor” as a result of “no need” (reference category). It was found by analysis that, compared with the reason of “no need”, the 45–59 age group and urban residents were evidently less likely to report “not see a doctor” due to “inconvenient traffic or no time to see doctor” or “distrust doctors or hospital service”. Moreover, cases with a health insurance or a better health status were less likely to report “not see a doctor” as a result of cost compared with “no need”.

**Table 3:** Multinomial logistic regression model for reasons for “not see a doctor” among Chinese aged≥45 years, 2015 (reference category is unmet need due to no need)

Factors	Inconvenient traffic or no time to see doctor	Distrust doctors or hospital service	Could not afford	Other
Sex				
Female	Reference	Reference	Reference	Reference
Male	1.145 (0.600–2.186)	1.004 (0.515–1.958)	1.270 (0.860–1.875)	0.906 (0.573–1.433)
Age group				
75+	Reference	Reference	Reference	Reference
60–74	0.407 (0.173–0.956)^*^	0.514 (0.209–1.262)	0.992 (0.535–1.841)	1.065 (0.545–2.080)
45–59	0.351 (0.135–0.913)^*^	0.245 (0.083–0.719)^*^	0.863 (0.440–1.696)	0.440 (0.202–0.960)^*^
Marital status				
Married	Reference	Reference	Reference	Reference
Others^a^	1.621 (0.830–3.165)	0.780 (0.336–1.811)	1.323 (0.862–2.032)	1.359 (0.836–2.209)
Hukou				
Rural	Reference	Reference	Reference	Reference
Urban	0.296 (0.098–0.890)^*^	0.709 (0.292–1.723)	0.582 (0.326–1.040)	1.049 (0.590–1.862)
Education				
No formal	Reference	Reference	Reference	Reference
Elementary school	0.685 (0.331–1.418)	1.275 (0.570–2.853)	0.897 (0.581–1.384)	0.490 (0.297–0.808)^**^
Middle school and above	1.053 (0.437–2.534)	1.358 (0.491–3.756)	0.665 (0.374–1.180)	0.672 (0.360–1.254)
Income				
Low	Reference	Reference	Reference	Reference
Middle	0.887 (0.411–1.913)	1.042 (0.463–2.345)	1.372 (0.865–2.176)	1.332 (0.782–2.268)
High	0.988 (0.474–2.058)	0.904 (0.393–2.079)	0.928 (0.576–1.497)	0.989 (0.561–1.743)
Access to pension^b^				
No	Reference	Reference	Reference	Reference
Yes	0.664 (0.352–1.256)	0.641 (0.324–1.267)	0.810 (0.539–1.218)	0.689 (0.433–1.098)
Access to health insurance^c^				
No	Reference	Reference	Reference	Reference
Yes	1.078 (0.391–2.970)	0.698 (0.251–1.940)	0.458 (0.267–0.786)^**^	0.984 (0.476–2.035)
Self-reported general health status				
Bad	Reference	Reference	Reference	Reference
Fair	1.187 (0.561–2.513)	0.751 (0.343–1.648)	0.441 (0.293–0.662)^***^	0.597 (0.365–0.976)
Very good/good	1.196 (0.526–2.720)	1.224 (0.540–2.774)	0.310 (0.188–0.511)^***^	0.550 (0.313–0.965)
Chronic illness				
No chronic disease	Reference	Reference	Reference	Reference
One chronic disease	1.215 (0.357–4.133)	0.186 (0.036–0.956)^*^	0.697 (0.317–1.532)	0.417 (0.185–0.940)^*^
Two chronic diseases	1.746 (0.519–5.870)	0.641 (0.185–2.221)	1.222 (0.569–2.622)	0.787 (0.365–1.697)
Three and more chronic diseases	2.195 (0.714–6.747)	1.629 (0.615–4.313)	1.766 (0.887–3.514)	0.957 (0.487–1.880)

^*^P < 0.05, ^**^P < 0.01, ^***^P < 0.001

^a^Include divorced or separated, widowed and single

^b^Include rural pension, urban residents’ pension, commercial pension, pension of the firms, pension program of the government or institutions and old age pension allowance or others

^c^Include urban employee medical insurance, urban and rural resident medical insurance, new cooperative medical insurance, urban resident medical insurance, private medical Insurance, government medical insurance

## Discussion

In this study, the proportion of “not see a doctor” among the elderly people aged ≥45 years in China was explored, and 11% of surveyed respondents reported “not see a doctor”. According to their definitions, “unmet healthcare demand” was contained in “not see a doctor”. Therefore, theoretically speaking, the proportion of unmet healthcare demands in Chinese aged ≥45 years should be less than 11%, while such figure was 3.9% in Irish aged ≥50 years [6], 15.6% in Swedes aged 45–80 years [17] and 16% in Korean aged ≥50 years [18]. It seemed like China had a relatively lower level of self-reported unmet demand compared with that in Sweden and Korea. However, the unmet healthcare demand between China and other countries could still hardly be compared due to the different definitions of the unmet demand in different studies.

Consistent with previous research, analysis in this study found higher levels of “not see a doctor” among those with older age [9, 19] and those with poorer health status [6, 9, 17]. Unlike other studies [6, 20], it was not found that females were more susceptible to “not see a doctor”. In addition, it was also discovered that, those who were married had reported a lower level of “not see a doctor”, while no relationship was discovered in the other literatures [6, 9]. It was evident that marriage could protect health through a mix of social and economic mechanisms, such as encouraging healthy behaviors and having health insurance [21]. Therefore, those who were married were more likely to see a doctor when they were ill. Moreover, other studies also confirmed that a lower income was notably associated with a higher odds of reporting unmet healthcare demands [6, 9, 10]. However, in this study, no association was found between income (as well as other enabling factors, such as whether access to health insurance and pension) and “not see a doctor”. The main reason was that the dependent variables were different between this study and others (“not see a doctor” vs “unmet healthcare demand”). Typically, the subjects in this study included those whose illness was not “necessarily treated”. Meanwhile, the results also showed that a majority of cases reported no need as the reason for their “not see a doctor”, and the proportion of this reason was higher in those with higher income, pension and health insurance than those with lower income and without pension or health insurance. In other words, people with higher income, pension and health insurance seemed more likely to report “not see a doctor” as a result of “no need”. Therefore, “whether need to see a doctor or not” was the key factor for producing different results between this study and others.

Apart from the cause of “no need”, factor associated with the healthcare system—cost—had also accounted for a large proportion of “not see a doctor”. In this study, approximately 15% of the surveyed respondents had reported “not see a doctor” due to cost, which was increased from 7.6% in 2013 [22]. Typically, “Whether access to health insurance” was a factor related to healthcare costs. Research showed that, the total healthcare costs were dramatically higher in those with health insurance than those without health insurance [23]. In other words, those without health insurance were more likely to have an unmet healthcare demand due to affordability issue, which was in keeping with our results.

For the affordability issue, the matter is not “it will cost”, but “cost whose money”. The binary model with negative log-log link function found there was no association between income and “not see a doctor”. However, to further analyze the specific cause distribution of “not to see a doctor”, the multinomial logistic regression analysis found there was still no association between income (as well as pension) and “not see a doctor”, but there was great significance between health insurance and “not see a doctor” due to “could not afford”. This reveals an interesting affordability issue——“not to see a doctor” is not a problem of lack of money, but who will pay money to see a doctor. If you have access to health insurance, most of the cost of seeing a doctor will be paid by the government or the health care provider. But if not, you have to pay your cost by yourself. This shows that having a guaranteed medical insurance plays an important role in the medical treatment of residents aged ≥45 years. In summary, it can be considered that, for the affordability issue, the matter is not “it will cost”, but “cost whose money”.

Nonetheless, several limitations should be noted in our study. Firstly, the analysis is based on a self-reported questionnaire, which is associated with potential recall bias. Secondly, the analysis is limited by the cross-sectional design, and the observed associations may not be causal. Thirdly, information about the knowledge regarding medicine and healthcare system among the study subjects is not collected in this study; as a result, it is difficult to investigate whether these factors are associated with “not see a doctor”.

## Conclusion

In conclusion, the analysis in this study finds approximately 11% of self-perceived “not see a doctor” in China. Moreover, the findings also highlight the relative importance of age, marital status and health status in the cause of “not see a doctor”. For China, except “no need”, the predominant cause of cost-related “not see a doctor” calls for a reduction of the healthcare fees and an increment of coverage of health insurance. Moreover, future studies on detailed status of unmet healthcare demands in China are warranted, including the unmet demand for the level of healthcare system, and whether the unmet demand remains unmet or is met at a later date.

## Data Availability

The datasets generated and/or analyzed during the current study are available in the CHARLS repository, charls.pku.edu.cn/en.

## Abbreviation

CHARLS: China Health and Retirement Longitudinal Study
